# Parental perspectives on the impact of screen time on the language skills of typically developing Indian children

**DOI:** 10.1590/2317-1782/20242023159en

**Published:** 2024-04-29

**Authors:** Febha Varghese, Sudhin Karuppali

**Affiliations:** 1 Department of Audiology and Speech Language Pathology, Kasturba Medical College, Mangalore, Manipal Academy of Higher Education - Manipal, India.

**Keywords:** Devices, Impact, Language, Parent, Screen-time

## Abstract

**Purpose:**

The overuse of screen-based devices results in developmental problems in children. Parents are an integral part of the children’s language development. The present study explores the parental perspectives on the impact of screen time on the language skills of typically developing school-going children using a developed questionnaire.

**Methods:**

192 parents of typically developing children between 6 and 10 years of age participated in the study. Phase 1 of the study included the development of a questionnaire targeting the impact of screen devices on language development. The questionnaire was converted into an online survey and was circulated among the parents in Phase 2. Descriptive statistics were performed on the retrieved data and a chi-square test was done to determine the association between the use of screen devices across all language parameters.

**Results:**

Parents reported television and smartphones to be the most used type of device, with a large proportion of children using screen-based devices for 1-2 hours per day. Most parents reported children prefer watching screens mainly for entertainment purposes, occasionally under supervision, without depending on them as potential rewards. The impact of screen-based devices on language skills has been discussed under the semantics, syntax, and pragmatic aspects of language.

**Conclusion:**

The findings of this study will help identify the existing trends in the usage of screen-based devices by children, thereby identifying potential contributing factors towards language delays. This information will also benefit in parental counselling during the interventional planning of children with language delays.

## INTRODUCTION

The development of technology over the past decade has drastically resulted in the increased use of electronic devices such as tablets, smartphones, computers, and gaming consoles, compared to traditional televisions which continue to be the most common mode of digital media viewing^(1)^. According to a US national survey, computer use (active screen time) among children between 6 and 12 years of age went from 18.2% to 29.2% between 1997 and 2003^(2)^, while children under 8 years spending over 2 hours/day with media, tripling their mobile usage from 2013 to 2017^(3)^. Due to the emergence of an intuitive touch-screen user interface on internet-enabled smart devices, the early use of technology has increased by 32% in the last two decades^(4)^. Watching television, streaming movies or shorter video content on mobile devices are primarily seen as passive activities resulting in increased screen time^(5)^. Screen time is the time spent viewing content, displayed and projected from active and passive screen media, that presents visual information on a two-dimensional display^(6)^. In contrast to earlier generations, current children are exposed to longer screen times, with the variety of available gadgets and applications significantly increasing in the market.

Video-based interactive platforms are found to influence social interaction in the global society^(7)^. A scoping review indicates negative effects on language development due to the amount of screen time and the early age of onset of screen viewing, with older age of onset viewing showing positive effects^(8)^. These positive effects include vocabulary expansion, exposure to diverse cultural and linguistic experiences, improving education, and keeping children engaged in a secured manner^(9)^. Qualitative and age-appropriate shows with clear educational objectives provide children an additional path to develop early language and literacy^(10)^. When combined with high-quality educational programming and co-viewing, the participatory aspect of these activities increases the children’s language competency^(8)^. Educational and commercial leaders encourage the use of digital technology by young children to better prepare them for success in this digital age^(11)^. However, health experts warn of possible detrimental consequences such as decreased attention span, fewer opportunities for verbal exchanges, hampered problem-solving, and diminished creativity^(12)^. Other negative effects may include having a decreased vocabulary^(8)^, delayed speech and language milestones^(8)^, impaired social interactions^(13)^, and reduced parent child interactions^(14)^. However, good quality of moderated screen exposure among older children (i.e., educational and co-viewing) appeared to be favorable for language development. In line with this, research has found no association between children’s language development and media exposure^(15,16)^.

Information technology has currently become a staple entity in every household, especially with parents who depend upon it for work or entertainment purposes. Unlike before, current children are exposed to screen devices right from birth due to the ease of access to technology. Children are sometimes encouraged to use these devices, especially when parents are doing important chores. Moreover, schools have started encouraging children to use such devices as part of their homework or assignments. This has resulted in children becoming over-dependent on such devices, especially when owning such devices nowadays has become more of a requirement than prestige. Since the beginning of COVID-19 pandemic in 2020, the demand for screen usage have escalated tremendously, with children spending a substantial amount of screen time during the day. Although it has been established that preschool years have exponential language growth, one might predict the detrimental effects of media to exist more in preschool than elementary school going children^(15)^. However, parents of older than younger children feel that their children have prolonged screen times with a negative influence on their child’s learning^(3)^, indicating concerns about screen usage to be prevalent in this age group as well. Having studied this older group will help provide definite recommendations for parents and caregivers about their children’s screen usage. Presently, there is a dearth of Indian studies done to understand the effect of screen viewing on the language skills in typically developing older children. The current study therefore attempts to explore the parental perspectives towards the impact of screen time on the language skills of typically developing school-going children between 6 and 10.11 years of age. The objectives of the study include: (1) developing a parental questionnaire to investigate the impact of screen time on the language skills of typically developing school-going children; and (2) administer the developed questionnaire on the parents of typically developing school-going children, and to analyze the obtained data using suitable statistical measures.

## METHODS

The current study followed a cross-sectional study design with a non-random convenience sampling method. The Institutional Ethics Committee approved the study protocol (IEC KMC MLR 05-2022/163) of Kasturba Medical College, Mangalore, Manipal Academy of Higher Education.

### Participants

The sample size for the current study was derived using the formula:


$$n\frac{Z^{2}\;pq}{d^{2}}\;$$ (1)

where Z = 1.96, at 5% level of significance, p= estimate proportion of population=60%, q= 1-p, d= 0.07 (7%).

The sample size for the current study was estimated from a parent article^(17)^. A total of 192 participants were included in the study. The participants comprised of parents of typically developing children between 6 and 10 years of age. The participants were recruited from various parts across Southern India. Table 1 shows the demographic details of the participants.

**Table 1:** The total number of parents under each age group

Age of the child (in years)	Total no. of parents
6-6.11	44
7-7.11	36
8-8.11	49
9-9.11	27
10-10.11	36
Total	192

Caption: no. = number

Prior to the conduction of the research, the participants were explained about the purpose of the study. The participants who fitted the selection criteria were recruited for the research. The inclusion criteria included parent/s of typically developing school-going children between 6 and 10.11 years of age. The exclusion criteria included parent/s of children with any type of communication disability, and parent/s of children who were unexposed to any screen devices.

### Procedure

The study was conducted in two phases. Phase 1 involved the development of the questionnaire, while phase 2 involved data collection and analysis.

#### Phase 1: Development of the questionnaire

The initial questionnaire (50 items) was constructed by reviewing the existing literature on effects of screen time on the language skills of children. This was content validated by three speech language pathologists with a minimum of 5 years of clinical and research experience in child language development. The authenticity of each of the items of the questionnaire was rated by the experts using a Likert rating scale (relevant, quite relevant, somewhat relevant, and not at all relevant). Along with the incorporations of the suggestions and comments by the three experts, a content validation index of 0.92 was obtained for the revised version of the questionnaire. The questionnaire targeted questions pertaining to type and use of screen-based devices, preference of screen-based devices and web-based platforms, screen-based dependency and level of supervision, and the impact of screen time on language skills. The responses of the items were in the form of Likert rating scale (always, often, sometimes, never), and multiple choices. The final questionnaire (28 items) was then ready for data collection.

#### Phase 2: Data collection and analysis

The items of the questionnaire were converted into an online survey (Google survey) with a common URL made available for the same. The survey began with a brief introduction to the purpose of the study, followed by an informed consent statement. Participants providing the consent went ahead providing their responses to each of the items in the survey. Each participant took an average of 10 minutes to complete the survey. The data collection was carried out between September - November 2022. The participant data were automatically protected in an online database accessible only to the authors of the study. The obtained data was subjected to statistical analysis using SPSS version 25.0. Descriptive statistics was used to determine the n (%) of the participants. In addition, the strength of association between the screen usage across all language parameters was measured using the Chi-square test.

## RESULTS

The current study aimed to investigate the parental perspectives towards the impact of screen time on the language skills of typically developing school-going children using a developed questionnaire. The results of the study are indicated below:

### Type and use of screen-based devices

**Figure 1:**
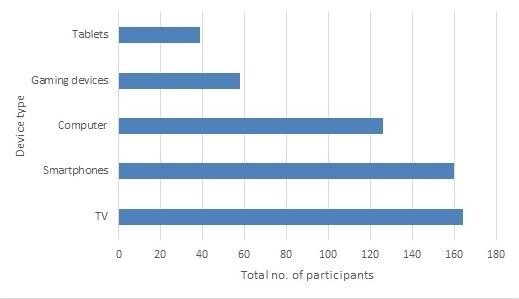
The types of screen-based devices used by children as reported by their parents

Figure 1 shows the types of screen-based devices used by children which were reported by their parents.

Considering the duration of screen usage, a maximum number of parents [117 (60.9%)] reported their children to use these devices for 1-2 hours per day, followed by 43 (22.4%) reported to use it for <1 hour, 31(16.1%) for 3-4 hours, and 1 (0.5%) for >5 hours. A total of 84 (43.8%) and 87 (45.3%) parents reported their children have used these screen-based devices often and sometimes respectively; while 13 (6.8%) reported to use them always, with 8 (4.2%) reported to have never used such devices at home. When considering the scheduling of screen time, most parents [90 (46%)] reported to not set any screen time limits for their children, with 71(37%) reporting to set limits with the child following the same, while 31 (16.1%) not following otherwise.

### Preference of web-based platforms

**Figure 2:**
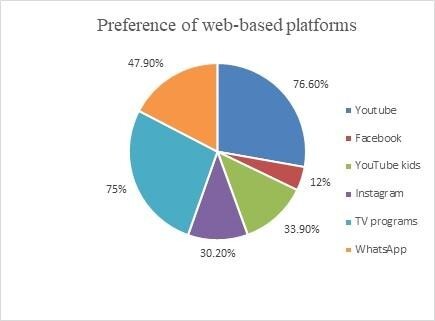
The types of web-based platforms used by children as reported by their parents

Two (1%) parents reported their children to be unexposed to web-based platforms. Figure 2 shows the web-based platforms preferred by children as reported by their parents.

It was observed that 139 (72.4%) parents reported their children used these devices specifically for entertainment, and 120 (62.5%) for learning-related purposes. A total of 93 (48.4%) parents reported using these devices for merely engaging the child by making phone calls, during food time, and for having social time.

### Screen-based dependency and level of supervision

Table 2 shows the level of dependency children has on screen-based devices and the level of supervision provided by parents during screen usage.

**Table 2:** The level of dependency children has on these devices and the level of supervision provided by parents during screen usage

	Always n (%)	Sometimes n (%)	Rarely n (%)	Never n (%)
Does your child use TV or any other gadgets under supervision?	53 (27.6)	117 (60.9)	NA	22 (11.5)
Does your child depend on these devices as a reward for doing an activity?	5 (2.6%)	62 (32.3%)	57 (29.7%)	68 (35.4)

Caption: NA = not applicable

#### Impact of screen time on language skills

The impact of screen time on the semantic (density and diversity of words), syntactic (comprehension and use of syntactic makers and sentences), and pragmatic (social use of language) aspects of the child’s language has been represented in Table 3. This includes the results obtained under each component of interest on the basis the Likert rating scale. The chi-square correlation between the usage of screen-based devices and the language parameters have been depicted in Table 4.

**Table 3:** Parental perspectives towards the impact of screen time on language skills

Items	Always n (%)	Most of the times n (%)	Sometimes n (%)	Never n (%)
Semantics				
Does your child like to learn new vocabulary after having used screen- based devices?	22 (11.5)	89 (46.4)	78 (40.6)	3 (1.6)
Does your child use all of his/her vocabulary on a frequent basis after having used screen-based devices?	29 (15.1)	78 (40.6)	77 (40.1)	8 (4.2)
Does your child use a variety of words to communicate after having used screen-based devices?	30 (15.6)	73 (38)	83 (43.2)	6 (3.1)
Does your child ask meanings of words after having used screen-based devices?	15 (7.8)	71 (37)	94 (49)	12 (6.3)
Syntax				
Does your child use sentences with 5 or more words frequently after having used screen-based devices?	64 (33.3)	90 (46.9)	35 (18.2)	3 (18.2)
Does your child address others by calling names instead of using ‘he’ ‘she’ ‘him’ ‘her’ after having used screen-based devices?	102 (53.1)	56 (29.2)	30 (15.6)	4 (2.1)
Does your child have difficulties understanding instructions at home after having used screen-based devices?	3 (1.6)	30 (15.6)	49 (25.5)	110 (57.3)
Does your child have difficulties understanding instructions at school after having used screen-based devices?	5 (2.6)	19 (9.9)	50 (26)	118 (61.5)
Do you find it difficult to understand your child after having used screen- based devices?	5 (2.6)	18 (9.4)	44 (22)	125 (65.1)
Does others find it difficult to understand your child after having used screen-based devices?	6 (3.1)	18 (9.4)	50 (26)	118 (61.5)
Pragmatics				
Does your child share information after having used screen-based devices?	18 (9.4)	62 (32.3)	90 (46.9)	22 (11.5)
Does your child initiate conversations with people after having used screen- based devices?	57 (29.7)	92 (47.9)	38 (19.8)	5 (2.6)
Does your child request other people after having used screen-based devices?	69 (35.9)	63 (32.8)	54 (28.1)	6 (3.1)
Does your child greet other people after having used screen-based devices?	100 (52.1)	53 (27.6)	36 (18.8)	3 (18.8)
Does your child thank other people after having used screen-based devices?	95 (49.5)	68 (35.4)	25 (13)	4 (2.1)
Does your child prefer playing in a group after having used screen-based devices?	50 (26)	106 (55.2)	33 (17.2)	3 (1.6)
Does your child understand and use higher language likes jokes and sarcasm after having used screen-based devices?	12 (6.3)	58 (30.2)	114 (59.4)	8 (4.2)
Does your child talk about activities at schools or friends’ homes after having used screen-based devices?	19 (9.9)	69 (35.9)	88 (45.8)	16 (8.3)
Does your child interact easily with other children and adults after having used screen-based devices?	28 (14.6)	120 (62.5)	36 (18.8)	8 (4.2)
Does your child finish the activities which he started after having used screen-based devices?	25 (13)	127 (66.1)	38 (19.8)	2 (1)
Is your child able to narrate stories after having used screen-based devices?	33 (17.2)	113 (58.9)	44 (22.9)	2 (1)

**Table 4:** Chi-square correlation between the usage of screen-based devices and the language parameters receiving a good level of significance (p<0.05)

Sl. No	Screen usage	Language component	Component parameter	Value
1.	Screen-based devices used by the child at home	Semantics	Asks meanings of words	X^2^(12)=28.362; p=.005
		Syntax	Frequent usage of 5 or more words	X^2^(12)=58.526; p=.000
			Using referent pronouns	X^2^(12)=34.626; p=.001
			Difficulties understanding instructions at school	X^2^(12)=56.102; p=.000
			Difficulties understanding instructions at home	X^2^(12)=23.911; p=.021
		Pragmatics	Initiate conversation	X^2^(12)=37.112; p=.000
			Requesting	X^2^(12)=42.595; p=.000
			Greeting	X^2^(12)=88.608; p=.000
			Thanking	X^2^(12)=50.220; p=.000
			Preference to play in group	X^2^(12)=42.152; p=.000
			Talking about activities at schools or friends’ homes	X^2^(12)=45.477; p=.000
			Finishing activities which he started	X^2^(12)=27.862; p=.006
			Narrating stories	X^2^(12)=39.815; p=.000
			Interact easily with others	X^2^(12)=25.467; p=.013
2.	The average hours the child using screen-based devices	Semantics	Asks meanings of words	X^2^(12)=37.231; p=.000
3.	The frequency of usage of screen-based devices	Syntax	Using referent pronouns	X^2^(9)=20.946; p=.013
			Difficulties understanding instructions at school	X^2^(9)=19.561; p=.021
		Pragmatics	Greeting	X^2^(9)=22.619; p=.007
			Interact easily with others	X^2^(9)=21.037; p=.012
4.	Limiting the screen usage	Semantics	Variety of words use to communicate	X^2^(9)=18.440; p=.030
		Syntax	Difficulties understanding instructions at school	X^2^(9)=33.922; p=.000
			Others find difficulties to understand child	X^2^(9)=48.707; p=.000
			Difficult to understand your child	X^2^(12)=56.420; p=.000
		Pragmatics	Requesting	X^2^(9)=22.127; p=.008
			Talking about activities at schools or friends’ homes	X^2^ (9)=17.794; p=.038
			Finishing activities which he started	X^2^ (9)=31.685; p=.000
5.	The web-based platforms used to watch	Semantics	Asks meanings of words	X^2^ (15)=30.142; p=.011
		Syntax	Frequent usage of 5 or more words	X^2^ (15)92.661; p=.000
			Using referent pronouns	X^2^ (15)=27.239; p=.027
			Others find difficulties to understand child	X^2^ (15)=37.665; p=.001
			Difficulties understanding instructions at home	X^2^ (15)=27.415; p=.026
		Pragmatics	Initiate conversation	X^2^ (15)=53.383; p=.000
			Requesting	X^2^ (15)=68.425; p=.000
			Greeting	X^2^ (15)=89.001; p=.000
			Preference to play in group	X^2^ (15)=77.140; p=.000
			Understands jokes and sarcasm.	X^2^ (15)=30.392; p=.011
6.	Watching screen devices under supervision	Semantics	New vocabulary learning	X^2^ (6)=15.650; p=.016
			Frequency of vocabulary used	X^2^ (6)=28.953; p=.000
			Variety of words use to communicate	X^2^ (6)=14.892; p=.021
			Asks meanings of words	X^2^ (6)=24.171; p=.000
		Syntax	Using referent pronouns	X^2^ (6)=19.243; p=.004
		Pragmatics	Requesting	X^2^ (6)=21.828; p=.001
			Preference to play in group	X^2^ (6)=14.554; p=.024
			Talking about activities at schools or friends’ homes	X^2^ (6)=31.165; p=.000
			Finishing activities which he started	X^2^ (6)=17.401; p=.008
			Narrating stories	X^2^ (6)=19.243; p=.004
7.	Dependency on screen-based devices as a potential reward	Semantics	Asks meanings of words	X^2^ (9)=18.492; p=.030
		Syntax	Frequent usage of 5 or more words	X^2^ (9)=20.467; p=.015
			Using referent pronouns	X^2^ (9)=25.151; p=.003
			Difficulties understanding instructions at school	X^2^ (9)=17.018; p=.048
			Difficulties understanding instructions at home	X^2^ (9)=44.057; p=.000
		Pragmatics	Initiate conversation	X^2^ (9)=18.827; p=.027
			Requesting	X^2^ (9)=36.650; p=.000
			Greeting	X^2^ (9)=40.269; p=.000
			Thanking	X^2^ (9)=32.440; p=.000
			Preference to play in group	X^2^ (9)=31.171; p=.000
			Talking about activities at schools or friends’ homes	X^2^ (9)=25.450; p=.003
			Interact easily with others	X^2^ (9)=24.540; p=.004
8.	Purposeful use of screen-based devices by the child	Semantics	Asks meanings of words	X^2^ (12)=36.196; p=.000
		Syntax	Frequent usage of 5 or more words	X^2^(12)=35.951; p=.000
			Using referent pronouns	X^2^(12)=22.902; p=.029
			Difficulties understanding instructions at home	X^2^(12)=27.190; p=.007
		Pragmatics	Initiate conversation	X^2^(12)=33.075; p=.001
			Requesting	X^2^(12)=22.694; p=.030
			Greeting	X^2^(12)=53.198; p=.000
			Thanking	X^2^(12)=46.200; p=.000
			Preference to play in group	X^2^(12)=27.507; p=.007
			Talking about activities at schools or friends’ homes	X^2^(12)=28.085; p=.005
			Finishing activities which he started	X^2^(12)=25.780; p=.012
			Interact easily with others	X^2^(12)=21.158; p=.048
9.	Sharing screen experiences with parents	Semantics	New vocabulary learning	X^2^ (9)=31.105; p=.000
			Frequency of vocabulary used	X^2^ (9)=25.988; p=.002
			Variety of words use to communicate	X^2^ (9)=25.148; p=.003
			Asks meanings of words	X^2^ (9)=44.930; p=.000
		Syntax	Frequent usage of 5 or more words	X^2^ (9)=18.754; p=.027
			Using referent pronouns	X^2^ (9)=28.802; p=.001
			Difficulties understanding instructions at school	X^2^ (9)=22.874; p=.006
			Difficulties understanding instructions at home	X^2^ (9)=33.822; p=.000
		Pragmatics	Initiate conversation	X^2^ (9)=19.093; p=.024
			Greeting	X^2^ (9)=24.639; p=.003
			Preference to play in group	X^2^ (9)=18.310; p=.032
			Talking about activities at schools or friends’ homes	X^2^ (9)=33.193; p=.000
			Narrating stories	X^2^ (9)=17.823; p=.037
			Interact easily with others	X^2^ (9)=19.093; p=.024

Caption: Sl. No.: Serial number

The below table only represents the chi-square values receiving a good level of significance (p<0.05) between the usage of screen-based devices and its corresponding language-component parameter. All other chi-square values between the two variables (screen-based devices and the corresponding language-component parameter) were found to possess a poor level of significance (>0.05).

## DISCUSSION

The present research investigates the parental perspectives towards the impact of screen time on the language skills of typically developing school-going children between 6 and 10 years of age using a developed questionnaire. The objectives of the study were to develop a parental questionnaire to explore their perspectives towards the impact of screen usage on the language skills of their children, followed by administering the same on the parents and analyzing the obtained data. The results are discussed as follows:

### Type and use of screen-based devices

As observed in the results of the current study, a large proportion of parents (83-85%) reported their children to be exposed to smartphones and televisions during their developmental years. Unlike yester years, smartphones have become an essential part of every household^(18)^ facilitating rapid and convenient access to the internet, similar to carrying a pocket computer, which is not just limited to making and receiving phone calls. Due to the size, mobility, streaming and interactive capabilities, and declining costs, mobile-based devices are increasingly replacing traditional media devices as the most preferred choice by children. The current televisions are manufactured with high-end features including internet connectivity, largely for entertainment and educational purposes. As observed in the current study, the preference for smartphones and smart TVs over tablets and gaming devices may be a consequence of the multifunctional use of smartphones which provides a better gaming experience, also considering the affordability and portability^(19)^ of the device. Computers and laptops have become an essential part of every household, especially due to the current technological era we live in. The COVID-19 global pandemic has resulted in a certain stratum of individuals continuing to work from home, especially those from the Information Technology (IT) sector^(20)^, with these individuals requiring constant access to laptops and/or computers. This has consequently resulted in a significant rise in screen usage reported in preschool children^(21)^. Furthermore, the Government of India has incorporated IT as part of the school curriculum, enforcing children to begin exploring IT devices at a very young age. With instant access to all such devices, it becomes challenging for parents to raise their children limiting their technological access. Although research states children aged between 0 and 5 years to spend an average of three hours every day using screens^(22)^, the current study observes a large proportion of parents reporting their children to spend 1-2 hours per day viewing screens. It has also been reported that no matter if the media usage is through a big screen or a tablet, the effects towards language development becomes detrimental^(23)^.

### Preference for web-based platforms

The current study revealed a large proportion (77%) of parents reporting their children to prefer using YouTube and watching television programs. YouTube which is a free video-sharing platform includes numerous channels sharing educational videos as well as unboxing or game videos created for children. It also includes videos of cartoons, animations, funny clippings, music, game tutorials, and “how to do” videos which have been identified as the most preferred videos of young children. YouTube kids is another important platform designed to make it safer and simpler for children to explore online videos. The videos on this platform are more filtered in nature, as this remains the primary concern of parents. A total of 34% of the participants reported using YouTube kids in the current study. A recent study revealed 37% of children to engage in YouTube viewing using shared mobile devices with family members, having a median YouTube duration of 61.2 min/day^(24)^. Research reports ‘Chu Chu TV nursery rhymes’, ‘Cocomelon Rhymes’, and ‘Action figures’ to be the most viewed content on YouTube and YouTube kids, with 96% of parents believing these programs to enrich the vocabulary of their children^(25)^. Programs such as ‘Dora The Explorer’ and ‘Blues Clues’, facilitated increased vocabulary and expressive language performance. Another widely used application for instant video access was WhatsApp, which facilitates the exchange of instant messages, pictures, video, and voice calls via an internet connection that has been installed on smartphones. Apart from the leading platforms such as YouTube and TV programs, nearly 48% of the participants in the current study reported preferring to use WhatsApp. The implementation of WhatsApp in academics has helped increase the student’s performance by sharing the received educational videos, thereby creating a more engaging environment, especially in an offline mode^(26)^.

### Screen-based dependency and level of supervision

The results of the present study showed a large proportion (88%) of parents reporting their children to watch or use these devices under supervision. In an era when media devices are increasingly designed for individual use, it becomes important to observe children’s screen time. Although it is known that children learn best from one-on-one interactions with adults, a joint media engagement is recommended with parents and children watching or sharing screen-time together, scaffolding children’s understanding by explaining situational meanings^(15,27,28)^. Most parents (46%) in the current study reported to not set screen time limits for their children, with their children viewing screens alone without parents co-viewing. The transition from watching television to using touchscreen devices makes it difficult for parents to monitor their children because of the smaller screen size, portability, and internet connectivity accessed through devices that these children use. For parents to get their work done, children are encouraged to use television and/or screen media even without supervision, especially when their parents are not at home. Parents do use media or technology to discipline or reward their children^(29)^ unlike the findings of the current study, wherein the children were not dependent upon screen-based devices as potential rewards for doing an activity. It should also be noted with caution that parents’ preoccupation with smartphones becomes the primary reason for children to have a technological preference at a very early age^(30)^ impacting the domains of language acquisition and communication^(31)^ Research does reveal proactive mothers to respond verbally to their children’s initiatives compared to idle mothers, thereby providing an enhanced language input for their children^(32)^.

### Impact of screen time on language skills

Although a proportion of parents in the current study reported positive outcomes from screen viewing, ranging from the child’s receptive and expressive skills towards certain components of vocabulary, syntax, and pragmatics (as indicated in Table 4), certain negative effects with screen usage have also been reported^(27,33,34)^. Recent research revealed Cocomelon’s YouTube channel helped introduce English vocabulary to children^(35)^. The current study observed screen usage in children resulted in limited requests for word meanings (55%), limited sharing of information (58%), reduced understanding and use of higher language likes jokes and sarcasm (64%), and limited talking about activities at schools or friends’ homes (54%). Effects on language usage can be directly attributed to the type of communication interaction the child is indulged in. Research concludes screen time (more specifically television time) to not completely displace non-screen activities (play, social interactions, and reading), and that most parents continued encouraging their children to engage in other activities throughout the day rather than only spending time in online media^(17)^. Contrastively, others indicated that active screen time with internet capabilities improved social interaction, building relationships, increasing communication, and supporting team-paly, cooperation, imagination, and developing curiosity^(36)^. With screen time inducing mixed effects on the child’s language development, it becomes difficulty to draw firm conclusions about the effect of screen viewing on language development^(37)^.

The current study helps explore the existing trends in the usage of screen-based devices by Indian children, which further helps identify certain contributing factors towards language delays seen in such children, thereby aiding in parental counselling of children with language delays. Since the physical presence of the parents during their children’s screen time cannot be ascertained, certain parental reports on the duration and preference of screen-based devices by the children is highly questionable. To overcome this issue, future studies may incorporate the information on the screen usage of children provided by other caregivers at home. The preference of the type of devices children used also reflects upon the availability of such devices at home, which could have impacted the results of the study. Moreover, since using screen-based devices have become mandated by certain schools for educational purposes, the usage of the same by the children is not purely based by choice.

## CONCLUSION

The results of the current study highlights both positive and negative effects of screen time on certain aspects of semantics, syntax, and pragmatics based on the type of screen-based devices used, the amount and frequency of usage, the web-based platform used, the extent of adult supervision and screen-sharing with parents, the dependency on screen-based devices and the purposeful use of these devices by children.

## References

[1] Braithwaite I, Stewart AW, Hancox RJ, Beasley R, Murphy R, Mitchell EA (2013). The worldwide association between television viewing and obesity in children and adolescents: cross sectional study. PLoS One.

[2] Browne D, Thompson DA, Madigan S (2020). Digital media use in children: clinical vs scientific responsibilities. JAMA Pediatr.

[3] Rideout V (2017). The common sense census: media use by kids age zero to eight..

[4] Goode JA, Fomby P, Mollborn S, Limburg A (2020). Children’s technology time in two US cohorts. Child Indic Res.

[5] Byrne R, Terranova CO, Trost SG (2021). Measurement of screen time among young children aged 0-6 years: a systematic review. Obes Rev.

[6] Suggate SP, Martzog P (2020). Screen-time influences children’s mental imagery performance. Dev Sci.

[7] Trost SG, Brookes DSK (2021). Effectiveness of a novel digital application to promote fundamental movement skills in 3- to 6-year-old children: a randomized controlled trial. J Sports Sci.

[8] Karani NF, Sher J, Mophosho M (2022). The influence of screen time on children’s language development: a scoping review. S Afr J Commun Disord.

[9] Balton S, Uys K, Alant E (2019). Family-based activity settings of children in a low-income African context. Afr J Disabil.

[10] Huston AC, Wright JC (1998). Television and the informational and educational needs of children. Ann Am Acad Pol Soc Sci.

[11] Straker L, Zabatiero J, Danby S, Thorpe K, Edwards S (2018). Conflicting guidelines on young children’s screen time and use of digital technology create policy and practice dilemmas. J Pediatr.

[12] Twenge JM, Campbell WK (2018). Associations between screen time and lower psychological well-being among children and adolescents: evidence from a population-based study. Prev Med Rep.

[13] Pandya A, Lodha P (2021). Social connectedness, excessive screen time during covid-19 and mental health: a review of current evidence. Front Hum Dyn..

[14] Wong RS, Tung KTS, Rao N, Leung C, Hui ANN, Tso WWY (2020). Parent technology use, parent-child interaction, child screen time, and child psychosocial problems among disadvantaged families. J Pediatr.

[15] Dore RA, Logan J, Lin TJ, Purtell KM, Justice LM (2020). Associations between children’s media use and language and literacy skills. Front Psychol.

[16] Lin HP, Chen KL, Chou W, Yuan KS, Yen SY, Chen YS (2020). Prolonged touch screen device usage is associated with emotional and behavioral problems, but not language delay, in toddlers. Infant Behav Dev.

[17] Singh SJ, Azman FNSM, Sharma S, Razak RA (2021). Malaysian parents’ perception of how screen time affects their children’s language. J Child Media.

[18] Nagata JM, Ganson KT, Iyer P, Chu J, Baker FC, Pettee Gabriel K (2022). Sociodemographic correlates of contemporary screen time use among 9- and 10-year-old children. J Pediatr.

[19] Radesky JS, Weeks HM, Ball R, Schaller A, Yeo S, Durnez J (2020). Young children’s use of smartphones and tablets. Pediatrics.

[20] Xiang S, Rasool S, Hang Y, Javid K, Javed T, Artene AE (2021). The effect of COVID-19 pandemic on service sector sustainability and growth. Front Psychol.

[21] Ribner AD, Coulanges L, Friedman S, Libertus ME, Hughes C, Foley S (2021). Screen time in the coronavirus 2019 era: international trends of increasing use among 3- to 7-year-old children. J Pediatr.

[22] Adams C, Kubin L, Humphrey J (2023). Screen technology exposure and infant cognitive development: a scoping review. J Pediatr Nurs.

[23] Sundqvist A, Koch FS, Birberg Thornberg U, Barr R, Heimann M (2021). Growing up in a digital world - digital media and the association with the child’s language development at two years of age. Front Psychol.

[24] Radesky JS, Seyfried JL, Weeks HM, Kaciroti N, Miller AL (2022). Video-sharing platform viewing among preschool-aged children: differences by child characteristics and contextual factors. Cyberpsychol Behav Soc Netw.

[25] Imaniah I, Fitria N, Dewi K, Zakky A (2020). You tube kids channels in developing young children’s communication skills in English: parents’ beliefs, attitudes, and behaviors. Int J Lang Educ Cult Rev..

[26] Mistar IB (2016). Students ‘perception on the use of WhatsApp as a learning tool in ESL classroom. J Educ Social Sci..

[27] Madigan S, McArthur BA, Anhorn C, Eirich R, Christakis DA (2020). Associations between screen use and child language skills: a systematic review and meta-analysis. JAMA Pediatr.

[28] Muppalla SK, Vuppalapati S, Reddy Pulliahgaru A, Sreenivasulu H (2023). Effects of excessive screen time on child development: an updated review and strategies for management. Cureus.

[29] Kabali HK, Irigoyen MM, Nunez-Davis R, Budacki JG, Mohanty SH, Leister KP (2015). Exposure and use of mobile media devices by young children. Pediatrics.

[30] Mustonen R, Torppa R, Stolt S (2022). Screen time of preschool-aged children and their mothers, and children’s language development. Children.

[31] Varadarajan S, Venguidesvarane AG, Ramaswamy KN, Rajamohan M, Krupa M, Christadoss SBW (2021). Prevalence of excessive screen time and its association with developmental delay in children aged <5 years: a population-based cross-sectional study in India. PLoS One.

[32] Snijders VE, Bogicevic L, Verhoeven M, van Baar AL (2020). Toddlers’ language development: the gradual effect of gestational age, attention capacities, and maternal sensitivity. Int J Environ Res Public Health.

[33] Collet M, Gagnière B, Rousseau C, Chapron A, Fiquet L, Certain C (2019). Case-control study found that primary language disorders were associated with screen exposure. Acta Paediatr.

[34] Rithipukdee N, Kusol K (2022). Factors associated with the suspected delay in the language development of early childhood in southern Thailand. Children.

[35] Anggraini PP, Apriliani NA, Supeni I, Handrianto C (2022). The use of the cocomelon youtube channel as a medium for introducing children’s english vocabulary. SAGA. J Engl Lang Teach Appl Ling..

[36] Wong YC, Ho KM, Chen H, Gu D, Zeng Q (2015). Digital divide challenges of children in low-income families: the case of Shanghai. J Technol Hum Serv.

[37] Lavigne HJ, Hanson KG, Anderson DR (2015). The influence of television coviewing on parent language directed at toddlers. J Appl Dev Psychol.

